# Altered relationship between subjective perception and central representation of touch hedonics in adolescents with autism-spectrum disorder

**DOI:** 10.1038/s41398-021-01341-7

**Published:** 2021-04-17

**Authors:** Irene Perini, Per A. Gustafsson, Kajsa Igelström, Brigita Jasiunaite-Jokubaviciene, Robin Kämpe, Leah M. Mayo, Johanna Molander, Håkan Olausson, Maria Zetterqvist, Markus Heilig

**Affiliations:** 1 Center for Social and Affective Neuroscience, Department of Biomedical and Clinical Sciences, Linköping University, Linköping, Sweden; 2 Center for Medical Image Science and Visualization (CMIV), Linköping, Sweden; 3 Department of Child and Adolescent Psychiatry, Region Östergötland, Linköping, Sweden; 4 Department of Psychiatry, Region Östergötland, Linköping, Sweden

**Keywords:** Autism spectrum disorders, Neuroscience

## Abstract

An impairment of social communication is a core symptom of autism-spectrum disorder (ASD). Affective touch is an important means of social interaction, and C-Tactile (CT) afferents are thought to play a key role in the peripheral detection and encoding of these stimuli. Exploring the neural and behavioral mechanisms for processing CT-optimal touch (~3 cm/s) may therefore provide useful insights into the pathophysiology of ASD. We examined the relationship between touch hedonics (i.e. the subjective pleasantness with which affective touch stimuli are perceived) and neural processing in the posterior superior temporal sulcus (pSTS). This region is less activated to affective touch in individuals with ASD, and, in typically developing individuals (TD), is correlated positively with touch pleasantness. TD and ASD participants received brushing stimuli at CT-optimal, and CT-non-optimal speeds during fMRI. Touch pleasantness and intensity ratings were collected, and affective touch awareness, a measure of general touch hedonics was calculated. As expected, slow touch was perceived as more pleasant and less intense than fast touch in both groups, whereas affective touch awareness was moderately higher in TD compared to ASD. There was a strong, positive correlation between right pSTS activation and affective touch awareness in TD, but not in ASD. Our findings suggest that altered neural coupling between right pSTS and touch hedonics in ASD may be associated with social touch avoidance in ASD.

## Introduction

To appreciate a caress might feel like a simple, effortless ability, but extensive neural processing occurs before the caress is “labeled” as welcomed, pleasant, and caring. Low-threshold mechanoreceptors detect information on modifications occurring on the skin and feed it to the brain through the spinal cord. Thickly myelinated Aß afferents promptly carry information about location, speed, and pressure of the caress while C-Tactile (CT) afferents convey speed-dependent information about the pleasantness of the touch. Tactile signals carried by both afferents integrate already at the dorsal horn^1^, and by the time they reach the cortex, they are further integrated with information about the state of the body, the context where the caress is being delivered, and importantly, about who is giving this caress. Multisensory integration is therefore a crucial aspect in processing information of social value and in regulating social behavior^2^.

According to the Diagnostic and Statistical Manual of Mental Disorders (DSM-5), autism-spectrum disorder (ASD) encompasses difficulties with social interaction and “restricted, repetitive patterns of behavior, interests, or activities” which may take a form of “hyper- or hyporeactivity to sensory input, or unusual interest in sensory aspects of the environment”^3^. Altered sensory processing in individuals with ASD is observed across all sensory modalities, including tactile, visual, and auditory^4^. Thus, multisensory integration might be of central relevance in characterizing the social difficulties observed in ASD^2^.

Clinical observations of sensory processing abnormalities, including altered tactile processing, have been documented since the original description of infantile autism by Leo Kanner^5^ and have since been corroborated by experiences from parents and teachers. These observations, although lacking experimental control, have contributed to the characterization of ASD. Altered tactile processing is commonly reported in ASD, but experimental studies that investigated affective touch by examining responses to selective CT-afferent stimulation are limited^6–8^. The CT-afferent system taps into both tactile and social domains and can potentially provide a useful tool for the understanding of the social difficulties observed in ASD.

Stimulation speed, force, and temperature which optimally activate CT-afferents are perceived as most pleasant^9^ and rewarding^10^, and have the qualities of a gentle caress. Specifically, CT-afferents’ mean firing frequency increases when the skin is stroked at slow speeds (~3 cm/s, range 1–10 cm/s)^11^, at light-pressure^12^ and when the probe used for the stroking is at skin temperature (~32 °C)^13^. This evidence suggests a link between the activity of those afferents and social communication^14^. At the brain level, Aß-targeted fast touch (e.g. ~30 cm/s), and CT-optimal slow touch trigger distinct event-related potential (ERP) profiles^15^, providing further support to their complementary contributions to touch appreciation^16^. Consistently, fMRI evidence shows that while the primary somatosensory cortex is highly activated by fast touch the right posterior superior temporal sulcus (pSTS) activates more to slow touch^17,18^; this activation correlates positively with pleasantness ratings following slow touch stimulation^19^. Meta-analysis of functional magnetic resonance imaging (fMRI) findings also points to a preferential activation of posterior insula to affective compared to discriminative touch^20^. However more recent evidence suggests activity in posterior insula for affective as well as discriminative touch^19,21^.

The literature on CT-optimal touch processing in ASD is limited, and available studies vary in methodology. In an fMRI study of 19 children and adolescents with ASD, Kaiser et al.^8^ investigated the processing of CT-optimal and CT-non-optimal touch by brushing at 8 cm/s on the arm and on the palm^8^, since CT-afferents are limited to hairy skin in humans^16^. In both ASD and typically developing subjects (TD), stimuli were perceived equally pleasant regardless of location. In another fMRI study in adults by Cascio et al. 2012, the authors explored pleasantness and roughness ratings for touch applied at 5 cm/s using different materials (soft cosmetic brush, plastic mesh and burlap). They found similar pleasantness and roughness ratings in ASD and TD across all materials. The soft brush was rated as most pleasant and least rough, while the plastic mesh was perceived as most unpleasant and rougher in both TD and in ASD individuals. These behavioral findings replicate previous pilot findings in a small sample of adults, in which soft brush was perceived as more pleasant than plastic mesh in both groups^6^. Overall, these studies suggest that at the behavioral level and in experimental settings, there are more similarities than differences between ASD and TD in pleasantness ratings of tactile stimuli, or that, alternatively, these differences might be subtle.

At a neural level, the right pSTS is of interest both in the context of autism research and in the domain of touch hedonics. Specifically, we grounded our investigation on pSTS on two key findings. The first relates to neural differences in ASD compared to TD. In one of the few available studies on affective touch processing in ASD, right pSTS activated differently to affective touch in children and adolescents with ASD^8^. The second finding relates to the role of pSTS in the context of touch hedonics in healthy individuals, showing that this region has a positive association with pleasantness ratings in TD individuals^19^. Building from this evidence, we aimed to expand the findings from Kaiser et al. and provide novel insights on the potential role of right pSTS in the processing of touch hedonics.

We investigated whether right pSTS shows a different relationship to touch hedonics in ASD compared to typically developing control subjects (TD). Specifically, we used the affective touch awareness score as a measure of touch hedonics instead of pleasantness ratings. As presented above, the available evidence in the literature does not show a clear difference in touch hedonics between ASD and TD using pleasantness ratings alone. As opposed to independent pleasantness ratings for slow and fast touch, affective touch awareness reflects the relative difference in touch pleasantness between CT-optimal and CT-nonoptimal speeds weighted by overall within-subject pleasantness^22^. It integrates perceived pleasantness for CT-mediated affective touch with general touch pleasantness. Therefore, affective touch awareness provides an overall pleasantness profile that can’t be represented by the individual scores. We demonstrate that ASD participants did not show a correlation between affective touch awareness and neural activity in right pSTS, whereas this correlation was strong in TD.

## Materials and methods

### Participants

Twenty-seven adolescents and young adults with ASD were recruited from the child and adolescent psychiatric clinic at Linköping University Hospital, Sweden. All ASD participants attended special school classes for pupils with ASD, in Linköping. Twenty-six age-matched typically developing control subjects (TD) were recruited through advertisements in schools and on Facebook. One ASD subject did not complete the scan and one TD did not meet inclusion criteria. Twenty-six ASD participants (22 males; mean age = 17; range 16–20; SD = 1; 4 females; mean age = 16.3; range 16–17; SD = 0.5) and twenty-five age-matched TD (22 males, mean age = 17.5; range 16–22; SD = 1.7; 3 females, mean age =17 range 16–18; SD = 1) were thus included in the behavioral and MRI analyses. (Table 1).

**Table 1:** Participant demographics.

Demographic characteristics	ASD *n* = 26 *n* (%)	TD *n* = 25 *n* (%)	Comparison statistic
Sex			
Male	22 (85%)	22 (88%)	
Female	4 (15%)	3 (12%)	
Age			
Males *m*, range (sd)	17.0, 16–20 (1.1)	17.5, 16–22 (1.7)	n.s.
Females *m*, range (sd)	16.3, 16–17 (0.5)	17.0, 16–18 (1.0)	n.s.
Handedness (EHI)			
*m* (sd)	55.8 (59.61)	80.5 (25.60)	*p* = 0.061
Parental highest education encoding			
University/college	11 (42%)	19 (76%)	n.s.
Theoretical high-school program	9 (35%)	5 (20%)	
Vocational high-school program	5 (19%)	0 (0%)	
Compulsary school	1 (4%)	1 (4%)	
Parent born in other country	6 (23%)	3 (12%)	n.s.
Current family structure			n.s.
Married/co-habitant	15 (58%)	15 (60%)	
Separated	11 (42%)	10 (40%)	
Single parent household	0 (0%)	0 (0%)	
ASD			
DSM-IV ASD diagnosis	100%		
Age at diagnosis *m*, range (sd)	13.9, 5.7–17.4 (2.7)		
Adult Autism-Spectrum Quotient *m* (sd)	26.5 (5.9)	11.4 (5.2)	*p* < 0.001
Beck Depression Inventory-II *m* (sd)	9.2 (6.8)	6.1 (5.4)	*p* = 0.062
Beck Anxiety Inventory *m* (sd)	10.1 (8.4)	3.8 (2.8)	*p* = 0.001
Social Touch Questionnaire *m* (sd)	38.2 (8.8)	26.4 (7.2)	*p* < 0.001
Social Responsiveness Scale–2 (TD = 24) *m* (sd)	77.2 (28.4)	13.4 (9.1)	*p* < 0.001
Five Health-Relevant Personality Traits Inventory			
Hedonic capacity *m* (sd)	3.0 (0.4)	3.3 (0.5)	*p* = 0.011
Antagonism *m* (sd)	2.5 (0.6)	2.4 (0.7)	*p* = 0.375
Negative affectivity *m* (sd)	2.3 (0.7)	1.9 (0.5)	*p* = 0.004
Alexithymia *m* (sd)	2.6 (0.5)	2.0 (0.5)	*p* < 0.001
Impulsivity *m* (sd)	2.4 (0.6)	2.5 (0.6)	*p* = 0.660
Psychiatric diagnoses^a^			
Depression	4 (15%)		
ADHD/ADD	13 (50%)		
Medications^b^			
SSRI	3 (12%)		
SSRI + atomoxetin	1 (4%)		
SSRI + bupropion	1 (4%)		
Antiepileptics		1 (4%)	
No medication	21 (81%)	24 (96%)	

^a^Participants could have more than one diagnosis.

^b^Medications at time of fMRI.

Exclusion and inclusion criteria were reviewed by a child and adolescent psychiatrist (author PG). Inclusion criteria for the ASD group were ASD diagnosis and age between 16 and 22 years (22 years being the upper limit for attending special school classes). Exclusion criteria were the presence of neurological disorders, intellectual disability, assessed according to DSM-5 criteria, present or past psychotic symptoms as part of the psychiatric history, insufficient knowledge of Swedish, fMRI contraindications, previous severe head injury, seizures, or other significant medical illness and premature birth (before 33 weeks of gestation). Participants received their clinical ASD diagnosis prior (mean age = 3.4; range 0.6–11; SD = 2.5) to this study based on DSM-IV/DSM-5 criteria. Thirteen participants with ASD were also diagnosed with attention-deficit/hyperactivity disorder (ADHD/ADD), and two of these also had depression. Two additional individuals with ASD were diagnosed with depression. Adolescents taking psychotropic medications were included (*N* = 6) provided that these were ongoing and unchanged for at least three months (Table 1). Fourteen individuals in the ASD group to whom central stimulants (CS) (*N* = 9) or melatonin (*N* = 5) were prescribed omitted medications during the day (stimulants) and the evening before (melatonin) of magnetic resonance imaging (MRI) session.

TD were included if they had no DSM Axis I or II disorder in the past year and were not taking any psychotropic medications. Adolescents meeting inclusion criteria were approached with oral and written information about the study. Participants (and parents, if the participant was less than 18 years of age) gave written informed consent. For participating in this study, the adolescents received a payment of 200 SEK (approximately 20 Euro) per hour. The sample size was based on minimum sample size requirements^23^, and depended on recruitment, eligibility criteria and by completion of the experimental session. We provide optimal sample size calculated using G*Power^24^ by entering a “minimum theoretically informative effect size”, based on effect-size estimates for common fMRI experimental designs (see Box 2 in^25^). Given a 2 × 2 factorial within-between subject design and assuming an effect size of Cohen’s f = 0.2, and an α = 0.05, a total sample size of 52 subjects is required to detect an effect with ≥80% power. The study was approved by the Linköping Regional Ethical Board (Dnr: 2016/224-32) and the study was carried out in agreement with the World Medical Association Declaration of Helsinki 1975, as revised in 2008. Clinical recruitment occurred from September 2016 to May 2018.

### Psychometric measures

Before the experimental part of the study, all the participants filled out questionnaires about their family, illnesses, medications, *Autism-Spectrum Quotient* (AQ)^26^ to evaluate the number of autistic traits; *Beck Depression Inventory-II* (BDI-II)^27,28^ for assessment of depression symptoms and their severity; *Beck Anxiety Inventory* (BAI)^29^ to obtain information about the anxiety symptoms, *Social Touch Questionnaire* (STQ)^30^ to assess individuals’ perception about social touch; *Social Responsiveness Scale–2* (SRS-2, filled-in by the parents)^31^ to quantitatively measure social responsiveness and social competence, core features in ASD; *Five Health-Relevant Personality Traits Inventory* (HP5 Inventory)^32^ to obtain detailed information about personality traits of the participants and *Edinburgh Handedness Inventory* (EHI)^33^ for the preferences of left or right hand in daily activities.

### Task

During the MRI experimental session, the participants engaged in three tasks, which included a social processing task, an affective picture processing task and a tactile stimulation task. This study focuses on the latter. Tactile stimuli were delivered over 9 cm on the dorsal part of the left forearm, using a soft, 70-mm wide, goat hair artist’s brush from proximal to distal direction and vice versa.

Stimuli consisted of manually delivered light tactile strokes at CT-optimal speed (slow touch, ~3 cm/s) and CT-non-optimal speed (fast touch, ~30 cm/s). Each stimulation trial lasted for 12 s, and the interstimulus interval (ISI) was 10–12 s. Stimulation trials were presented 5 times per velocity and in three consecutive runs, for a total of 15 stimulation trials per velocity. Stimulation order was counterbalanced within/between runs and session. Stimuli were delivered by two trained female experimenters (one per participant) guided by audio scripts. During each run and for four stimulation trials, participants were asked to rate the pleasantness and intensity of slow and fast touch, on a visual analog scale (VAS), with scale anchors “unpleasant-pleasant” and “not intense-intense”, respectively. The VAS scale was presented on the screen after the stimulation trial, with the cursor initially presented centrally, and participants could move the cursor left and right using buttons positioned in their right hand. For each participant a total of 3 pleasantness and 3 intensity ratings per velocity were collected. The scale was converted to the range −10 to +10. For calculation of the affective touch awareness score, pleasantness and intensity ratings were converted to the range 0 to 10, as in Croy et al.^22^.

### MRI session and data acquisition

Before the hour-long fMRI session, participants underwent a training session in an MR simulator (Magnetic Resonance Simulator (PST MR Simulator System, BlindSight GmbH, Schlitz, Germany). During the training session, participants were habituated to the MRI environment and trained to lie still via feedback from a motion tracking system positioned around their head (MoTrak Head Motion Tracking System, Psychology Software Tools, Sharpsburg, PA, USA). In addition, the participants received instructions and did a trial run of the task. The training session was followed by an fMRI session at the Center for Medical Image Science and Visualization (CMIV), Linköping University Hospital. Imaging was performed using a Siemens Prisma 3 Tesla MR scanner (Siemens, Munich, Germany) equipped with a 64-channel head-coil. Blood oxygen-level-dependent (BOLD) data were acquired with an echo-planar imaging (EPI) sequence: TR = 901 ms; TE = 30 ms; flip angle = 59°; matrix size 64 ×64; field-of-view = 192 × 192 mm; voxel-size = 3 mm isotropic. Three functional task-runs were collected, and each run lasted for about 5 minutes. A resting state scan was acquired after the T1-weighted scan, while participants were told to look at a fixation cross positioned in the middle of the screen and used for functional parcellation (Supplemental Material). A high-resolution 3D T1-weighted Turbo Field Echo scan was acquired before the EPI data acquisitions TR = 2300 ms; TE = 2.36 ms; flip angle = 8°; matrix size = 288 × 288 mm; field-of-view = 250 ×250; voxel resolution = 0.9 isotropic.

### MRI data preprocessing and analysis

Functional MRI data were preprocessed and analyzed using the Analysis of Functional Neuro Images (AFNI) software v16.2.12^34,35^. Each EPI volume was registered to the volume with the minimum outlier fraction (using the AFNI outlier definition). Functional images were then warped to MNI template space using linear and non-linear transformations via AFNI’s @SSwarper function^36^. Nuisance effects due to head motion (estimated from the motion correction procedure) were accounted for by adding the motion parameters as regressors of no interest in the main regression. A motion censoring threshold of 0.3 mm per TR was implemented in combination with an outlier fraction threshold of 0.1. Volumes violating either of these thresholds were subsequently ignored in the time-series regression.

A general linear model (GLM) analysis was performed to capture differences across conditions (i.e. slow and fast touch). A unique input stimulus function was defined for each task period. Input stimulus functions were convolved with the AFNI gamma hemodynamic response function to yield regressors for the GLM. Whole-brain, voxel-wise GLM statistical analysis was carried out on the BOLD time-series data using 3dDeconvolve. Two regressors were created, one for slow and one for fast stimulation periods. In addition, regressors modeling motor presses and rating periods were included. A factorial 2x2ANOVA with “speed” (levels: slow, fast) as a within-subject factor and “group” (levels: ASD, TD) as a between subject factor was performed using the AFNI function 3dMVM^37^. Whole-brain results were thresholded at a per-voxel *p* = 0.002, cluster corrected at alpha = 0.05, according to current AFNI guidelines^38^.

We then investigated the correlation between affective touch awareness and right pSTS activity during slow compared to fast touch using three complementary approaches. First, to expand on the investigation by Kaiser et al.^8^ we defined the right pSTS mask anatomically using the Desikan–Killiany cortical atlas and extracted ß-scores during slow and fast touch^8^. Given that anatomically based parcellations do not necessarily guarantee functional overlap^39^, we tested whether the results from the first approach could be replicated when the region was defined on the individual level using ICA-based functional parcellation of the STS (Supplemental Material). In the third approach, at the within-group level, we performed a correlation analysis between brain response to slow and fast touch and the affective touch awareness score using AFNI function 3dttest + + with affective touch awareness scores as a covariate of interest (per voxel *p* < 0.005, cluster corrected at alpha = 0.01). For all three approaches the difference of extracted ß-scores for slow and fast touch “ß-scores (slow-fast)”, were correlated to affective touch awareness scores using Statistical Package for the Social Sciences (SPSS) version 25. Using Fisher r-to-z transformation we compared Pearson’s correlation coefficients between groups.

We tested the robustness of significant correlations by applying statistical bootstrapping, where pSTS ß-values from the first-level fMRI analysis and affective touch awareness scores were sampled randomly with replacement (100,000 bootstrapping iterations)^40,41^. Resulting correlation distributions in which the middle 95% did not overlap with zero were considered robust. Further, we tested the reliability of between-group correlations differences by computing an additional distribution (“difference distribution”) that included the difference in correlation coefficients between groups for each round of bootstrapping.

### Behavioral data analysis

Pleasantness and intensity touch ratings were analyzed using a repeated measures ANOVA with “speed” (levels: slow, fast as a within-subject factor and “group” (ASD, TD) as a between subject factor. Sex, comorbid diagnoses of ADHD/ADD and depression, and CS medication were included as covariates of no interest in the statistical analysis. Post hoc tests were performed using Paired Samples Student’s t-tests. More exploratively, the relationship between pleasantness and intensity ratings was investigated using Pearson’s correlations (Supplemental Material). ANOVAs, t-tests and correlations were calculated using SPSS version 25.

Affective touch awareness was calculated as the difference in pleasantness ratings for slow and fast touch weighted by overall within-subject pleasantness ratings^22^. In the original behavioral study, they used five different speeds, whereas in our fMRI study we have used two. Thus, affective touch awareness = [x̅pleasantness (slow) - x̅pleasantness (fast)] * [Σ(x̅pleasantness (slow); x̅pleasantness (fast))/2]. We compared affective touch awareness scores as well as distributions between groups. Our hypothesis was that the ASD group would show decreased hedonic discrimination between slow and fast touch, resulting in lower touch awareness scores and smaller distribution, clustered more towards zero. Analyses are reported with and without outliers, with outliers defined as scores above or below two standard deviations from the mean value. Scores were non-normally distributed (*p* = 0.003) and between-group comparisons were performed using the Mann–Whitney *U* test using SPSS and one-tailed Kolmogorov–Smirnov test using MATLAB 9.6 (www.mathworks.com). Finally, and to address replication of previous findings^22^, we performed a correlation between affective touch awareness and AQ scores using SPSS.

## Results

### Pleasantness and intensity ratings

**Fig. 1:**
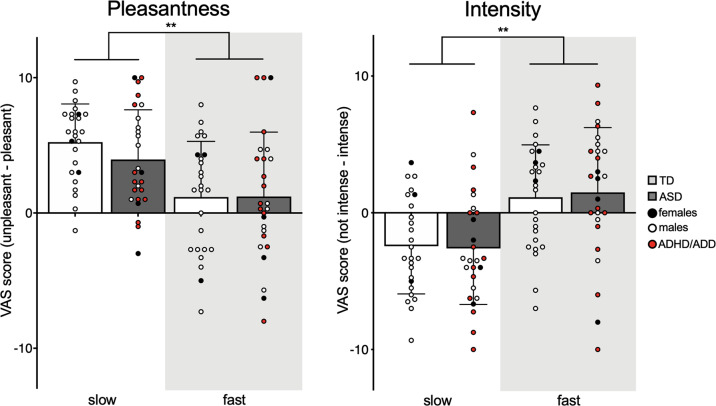
Pleasantness and intensity ratings. Slow brushing was perceived as more pleasant and less intense than fast brushing in both groups [ASD (*N* = 26) nd TD (*N* = 25)]. Sex, ADHD/ADD and depression comorbidities and CS medication were included as covariates. **indicate *p* < 0.001. Error bars indicate standard error of the mean (SEM).

We identified no difference in pleasantness or intensity ratings between groups. In both groups, slow touch was perceived as more pleasant and less intense than fast touch, replicating previous findings. For pleasantness ratings, a significant main effect of speed was observed [*N* = 26 ASD, *N* = 25 TD; *F*(1, 46) = 23.3, *p* < 0.001, *η*^*2*^*p* = 0.34], with slow touch rated as more pleasant than fast touch (*t* = 5.94*, p* < 0.001*; M*(slow TD) = 5.24, SD = 2.82; *M*(slow ASD) = 3.95, SD = 3.67; *M*(fast TD) = 1.18, SD = 4.12; *M*(fast ASD) = 1.21, SD = 4.77). No interaction or group effect was observed (all ps > 0.1). A significant main effect of speed was also detected for intensity ratings [*N* = 26 ASD, *N* = 25 TD; *F*(1,46) *=* 15.3*, p* < 0.001*, η2p* = 0.25], with fast touch rated as more intense than slow touch (*t* = −5.3*, p* < 0.001*; M*(slow TD) = −2.44, SD = 3.49; *M*(slow ASD) = *−*2.6, SD = 4.1; *M*(fast TD) = 1.13, SD = 3.83; *M*(fast ASD) = 1.49, SD = 4.74). Similar to ratings of touch pleasantness, no interaction or group effect was observed (all ps > 0.4) (Fig. 1).

### Affective touch awareness

**Fig. 2:**
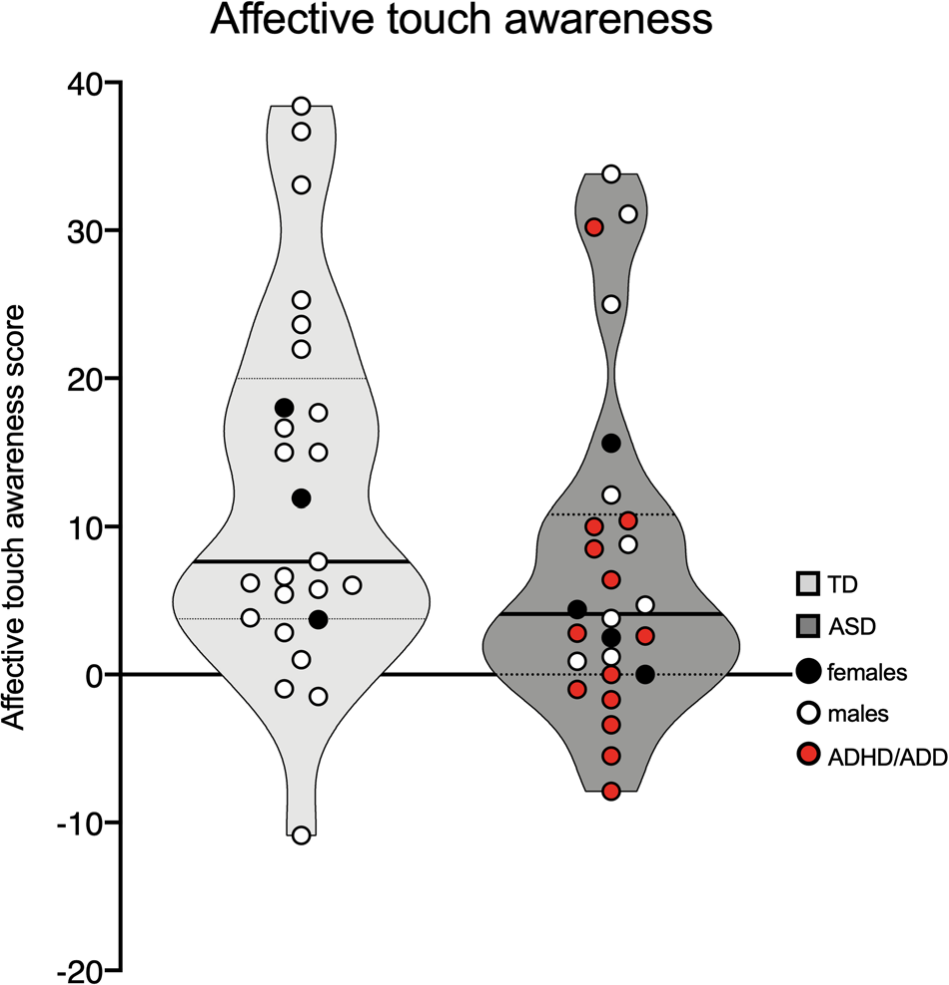
Affective touch awareness scores. Individuals with ASD (*N* = 26) had marginally lower scores compared to TD (*N* = 25) (*p* = 0.08).

There was a trend for affective touch awareness scores to be lower in ASD compared to TD (*U* = *232*, *p* = 0.08), and their distribution was narrower (*p* = 0.08, *D* = 0.30, alpha = 0.05, one-sided) (Fig. 2). When outliers (three in the ASD group and one in the TD group) were removed, this trend reached significance (*U* = 165, *p* = 0.02) and score distribution was significantly lower compared to TD (*p* = 0.036, *D* = 0.36, alpha = 0.05, one-sided). Affective touch awareness scores did not correlate with AQ scores in either groups (*ps* > 0.2).

### fMRI

In both groups we observed BOLD activity in typical regions involved in tactile processing, including primary and secondary somatosensory cortices and posterior insula (Tables S1). Compared to slow touch, fast touch resulted in increased primary somatosensory activation (Area 3b), whereas the opposite contrast (slow minus fast) showed increased activation in Area 2 (for all regions see Table 2). No between-group difference was identified.

**Table 2:** Activations associated with the whole-brain, gray matter 2 × 2 ANOVA analysis, expressed by peak scores in MNI space coordinates (*x*, *y*, *z*).

Analysis	Region	MNI coordinates			
		*x*	*y*	*z*	voxels
Main effect of speed					
Slow > Fast					
	Postcentral gyrus (Area 2)	64	−14	34	101
		−44	−35	46	9
		−44	−38	49	9
		−59	−26	40	8
	Superior frontal gyrus (Area 6_anterior)	28	−8	52	9
	Temporoparietal Occipital junction	58	−59	7	6
Fast > Slow	Postcentral gyrus (Area 3b)	37	−29	70	44
	Parietal Operculum (OP3)	37	−17	19	10

Z-scores survived significance threshold (*p* < 0.002, cluster corrected alpha < 0.05).

**Fig. 3:**
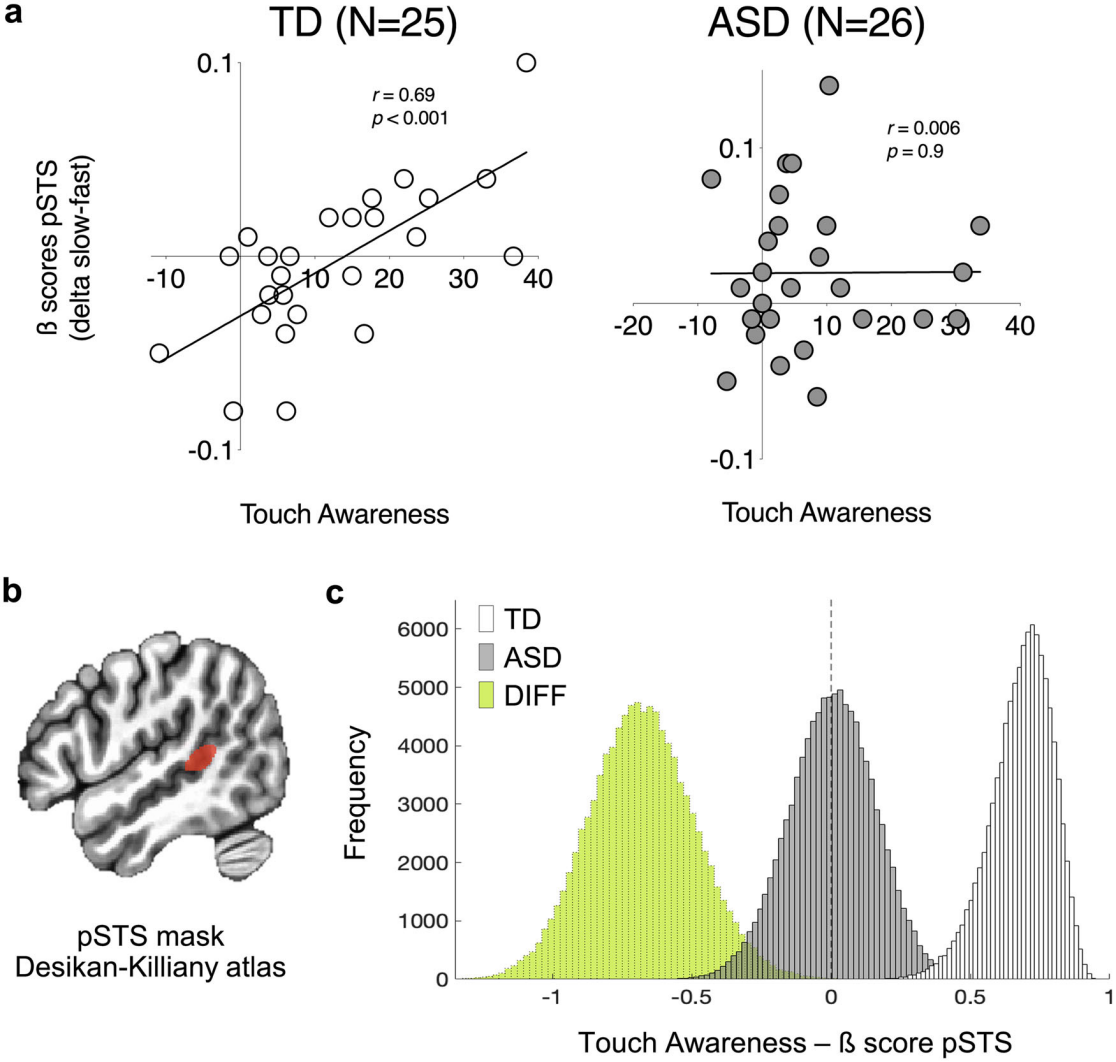
Brain findings. **a** Scatterplots showing Pearson’s correlations between the difference in ß-values for slow and fast touch and affective touch awareness scores in TD (*r* = 0.69, *p* < 0.001) and ASD (*r* = 0.006, *p* = 0.9). **b** Location of right pSTS mask, anatomically defined using the Desikan–Killiany atlas. **c** Histograms from 100,000 bootstrap iterations computing correlations between right pSTS response and affective touch awareness correlations. Frequency distribution for bootstrap iterations for ASD (gray, 95% CI [−0.30, 0.30]), for TD (white, 95%CI [0.43, 0.86]), and for their respective difference (green, 95% CI [0.29, 1.04]). The distribution of the difference appears to be reliably smaller than zero, driven by reliable increase in correlation in TD, which was not observed in ASD.

We found differences in the relationship between right pSTS and affective touch awareness among the two groups. In TD, but not in ASD, there was a strong positive correlation between the difference of ß-scores for slow and fast touch in right pSTS and affective touch awareness (Fig. 3a) using all three approaches (cf. Methods). More specifically, a positive correlation between affective touch awareness and ß-scores (slow-fast) was identified when defining right pSTS anatomically using the Desikan–Killiany atlas in TD but not in ASD (TD *r* = 0.69, *p* < 0.001; ASD *r* = 0.006, *p* = 0.9; Fig. 3a, b). Correlation scores were significantly different (*z* = 2.82, two-tailed *p* = 0.002) and the distributions of Pearson’s *r* derived during bootstrapping permutations were different than zero with > 95% confidence in TD (CI [0.43, 0.86]) but not in ASD (CI [−0.30, 0.30]). The distribution of correlation differences was greater different than zero with > 95% confidence, driven by a reliable positive correlation in TD, which was not seen in ASD (Fig. 3c).

When the right pSTS region was localized in individual subject using functional connectivity-based parcellation of resting state data, similar results were obtained (TD *N* = 21, *r* = 0.59, *p* = 0.004; ASD *N* = 24, *r* = 0.07, *p* = 0.7; *z* = 1.89, two-tailed *p* = 0.058) (see Fig. S1 and Supplemental Material). Finally, in TD, a positive correlation performed at whole-brain, gray matter level was observed between affective touch awareness scores and a cluster in the same location in right pSTS in response to slow versus fast touch (MNI 58, −35, 1; per-voxel *p* = 0.005, cluster corrected at alpha = 0.05, voxel size = 9; *r* = 0.81, *p* < 0.001). In ASD, no significant correlations were identified between affective touch awareness and brain activity for slow versus fast touch. The correlational results were unaffected by removal of outliers [Desikan–Killiany mask (TD *r* = 0.59, *p* = 0.002; ASD *p* = 0.9), connectivity-based mask (TD *r* = 0.46, *p* = 0.04; ASD *p* = 0.8), whole-brain-analysis based mask (TD *r* = 0.76, *p* < 0.001; ASD *r* = −0.23, *p* = 0.3).

## Discussion

We have identified a robust correlation between affective touch awareness and neural activity in right pSTS in TD. There was no such correlation in ASD. The lack of the brain-to-behavior coherency between right pSTS activity and touch awareness in ASD might result in a reduced ability to appreciate the properties of affective touch.

STS is a highly heterogeneous region involved in multisensory integration^42^ and social cognition^43^. Its posterior portion correlates with affective touch pleasantness in TD participants^19^, and has been recently suggested as a relevant hub, together with the temporoparietal junction, in coordinating the complexity of social dynamics^44^. Several studies have shown anatomical and functional differences in right pSTS in ASD compared to TD. These differences include decreased gray matter density^45^, decreased or absent STS activity during observation of affective touch^46^, processing of biological motion^47^, mentalizing initiated by animated shapes^48^, and voice processing^49^. Even in TD individuals, a negative correlation between the amount of autistic traits and right posterior STS activation has been observed in response to affective touch^50^. Specifically to the context of affective touch processing, Kaiser et al.^8^ show decreased activity in pSTS to slow touch in children and adolescents with ASD. We did not replicate the overall between-group brain findings presented in Kaiser’s et al.^8^. This is possibly due to population sample differences and tactile stimuli used. In Kaiser et al.^8^, the age range was wider than ours (i.e. 6-20) and they used different tactile stimuli (i.e. hairy vs glabrous skin). Both speed and location are similar methods in differentiating between CT-optimal and CT-nonoptimal stimulation, so we believe that the age range might be the most relevant aspect behind the inconsistency. That said, while the group effect did not replicate at whole-brain level, our correlational results are consistent with the findings by Kaiser et al.^8^. They also expand on their findings to suggest a role of right pSTS in the appreciation of the hedonic value of CT-“loaded” tactile stimulation, which might be altered in ASD. Thus, this finding provides novel insights in the understanding of the neurobiology of touch hedonics in autism.

We identified similar neural activation in ASD and TD to CT-optimal and CT-non-optimal touch. Our fMRI results replicated previous fMRI findings, with activations in primary and secondary somatosensory cortices and posterior insula to both fast and slow touch^19–21^. In addition, we found increased primary somatosensory cortex activity to fast compared to slow touch (Area 3b), which also replicated previous findings. For the slow minus fast comparison, we identified activity in another portion of the primary somatosensory cortex, corresponding to Area 2, which has to our knowledge not been documented before. Future studies might address whether this finding relates to developmental aspects of neural processing of affective touch. Furthermore, for the slow minus fast contrast, we found no posterior insula activation. The lack of posterior insula activation is inconsistent with earlier studies on affective touch in adults^51–53^. However, there is a growing body of evidence suggesting the engagement of insular cortex for both CT-optimal and CT-non-optimal touch, challenging the idea of posterior insular cortex as cortical target of CT-optimal touch^18,19,21,50^.

At the behavioral level, CT-optimal touch was found to be more pleasant and less intense than CT-non-optimal touch in TD as well as in ASD. This finding robustly replicates extensive evidence in adults and across the lifespan^54^. No difference between ASD and TD in pleasantness or intensity scores was identified, also replicating previous findings^6–8^ (see also correlational results in Supplemental Material). In order to achieve a comprehensive profile of tactile pleasantness, we calculated the affective touch awareness score, which includes information about the relative difference in tactile pleasantness perception between CT-optimal and CT-non-optimal speeds, while taking into account overall pleasantness rating values. First introduced by Croy et al. 2016, it was explored in a transdiagnostic group of psychiatric patients and age-matched TD. The authors found a significant negative correlation between affective touch awareness scores and AQ scores, indicating that the higher autistic traits the lower the appreciation of CT-targeted touch. In our study, affective touch awareness was marginally lower in ASD and the distribution of scores distribution in ASD was narrower, and clustered more closely towards zero (both scores significantly lower after removal of outliers), suggesting poorer discrimination between pleasantness for slow and fast speeds in the ASD group.

This study has some limitations. Our study does not replicate the negative AQ to affective touch awareness correlation reported by Croy et al. in a large sample of transdiagnostic patients and TD. The discrepancy between these findings might be related to several methodological differences, which relate to the different scopes of the two studies. Differently from our study, Croy et al. had a larger age span (age range 21–70), more tactile speeds (5 speeds), and a larger sample size (*N* = 69 TD and *N* = 70 patients)^22^. Although we used fewer speeds, the range of affective touch awareness scores and between-group findings are comparable to Croy et al. 2016. Therefore, the lack of replication is probably related to our lower sample size and with our age group, which included mostly late adolescents. A potential confounding factor that we could not address in the current study is intelligence quotient (IQ). While none of our participants presented with intellectual disability according to DSM-5 criteria, we cannot exclude that heterogeneity in IQ scores might explain some of the observed results. Our ASD group had high comorbidity with ADHD/ADD (*N* = 13). Children are often diagnosed with both ASD and ADHD^55^ and co-occurrence of these conditions has been recognized in the DSM-5^3^. Moreover, it has been shown that individuals with one disorder might show some traits of other conditions^56^, and some findings suggest that children diagnosed with ASD display the comparable levels of impulsivity as the ones with ASD/ADHD^55^. Pleasantness and intensity ratings were not confounded by ADHD/ADD diagnosis.

In summary, we provide evidence of a robust correlation between affective touch awareness and neural activity in right pSTS that is present in TD but absent in ASD. Our result supports the view that ASD is associated with a difference in neural processing in right pSTS^8^, a finding that may have diagnostic or therapeutic implications.

## Supplementary information

Supplemental Material
