# Supplementary material for: Altered relationship between subjective perception and central representation of touch hedonics in adolescents with autism-spectrum disorder
Source: Transl Psychiatry. 2021 Apr 17;11:224. doi: 10.1038/s41398-021-01341-7 (PMC8053196; doi:10.1038/s41398-021-01341-7)
Supplement: Supplementary file 1 — Supplemental Material [file 41398_2021_1341_MOESM1_ESM.docx]

**SUPPLEMENTAL MATERIAL**

**SUPPLEMENTAL MATERIAL: METHODS**

**Resting State**

Resting state data were used to define the coordinates of individual-level ROIs as a complement to the volumetric atlas-based approach. We used independent component analysis (ICA) to parcellate the right superior temporal sulcus (STS) into spatially independent sources, followed by dual regression to obtain individual coordinates for the functional units corresponding to the Desikan-Killiany atlas mask used in the initial analysis. An advantage with this approach is that it is potentially less affected by the great spatiotemporal variability that exists in large parts of the posterior temporal lobe e.g.^1^.

**Localized ICA**

The STS was parcellated using localized probabilistic ICA on the group level^2^, at a dimensionality of 20 as previously described^3,4^. This allows a fine-grained parcellation that is free of influences from dominant signals in other regions^3-6^.

A mask covering the entire right STS (Fig. S1b, left section) was constructed by converting the aparc.a2009s label (“rh.S_temporal_sup”) in the Destrieux atlas to a NiFTi volume using the cvs_avg35_inMNI152 standard surface in Freesurfer https://surfer.nmr.mgh.harvard.edu;^7,8^. The Destrieux atlas is a parcellation of the cerebral cortex into gyri and sulci based on the curvature of the surface, in which sulci correspond to regions not visible on the pial surface^7^. The resulting volumetric mask was blurred with a Gaussian kernel (6 mm FWHM), then binarized and resampled to the resolution of the functional scans. This smoothed mask was slightly dilated compared to the atlas, to ensure good coverage of the sulcus in all individual subjects.

An ICA dimensionality of 20 was chosen because it was previously found that independent components were stable at and above this model order in the temporoparietal region^3^. ICs were thresholded at *z* = 2.3 (mixture-model threshold of *P* < 0.5) and their overlap with the pSTS mask of the Desikan-Killiany atlas was quantified. The group-level component that showed the greatest overlap with the pSTS mask is shown in Figure S1b (right section; *cf.* Fig. 3b).

**Dual regression**

The pSTS IC was subjected to a dual regression analysis to derive an individual spatial map for each participant^9^. In this process, the group-average spatial maps are first used as spatial regressors in a multiple regression, resulting in a set of subject-specific time series. Second, those time series are regressed into the individual fMRI data, resulting in a set of subject-specific spatial maps^9^. Finally, individual peak coordinates in MNI space were identified using 3dClusterize (AFNI) and used to define the center of individual ROI masks (8 mm diameter). The average ß scores within the subject-level ROIs were extracted for slow and fast conditions.

**SUPPLEMENTAL MATERIAL: RESULTS**

**Pleasantness and intensity ratings**

We observed no relationship between pleasantness and intensity ratings to slow touch in both groups, however in ASD a significant association was identified for fast touch. For slow touch, the correlations between intensity and pleasantness ratings were negative but not significant (*ps* > 0.1). We found a significant negative correlation in the ASD group for fast touch (*r* = -0.51, *p* = 0.008; TD *p* = 0.9). While the relationship between pleasantness and intensity ratings is not often investigated, it might be useful in understanding the interplay of intensity and affective aspects of tactile information. In both groups, for touch with strong CT-afferents input, no relationship was observed between pleasantness and intensity ratings, suggesting that speed might be the most relevant feature for pleasantness evaluation. In the ASD population, we identified a significant negative correlation between pleasantness and intensity ratings to fast touch. While this is an exploratory finding, it suggests a relationship between perceived pleasantness and intensity in the ASD group for tactile stimulations of more “discriminative” nature. Thus, while for controls speed seems to be overall the most relevant feature in the evaluation of pleasantness of touch, in ASD intensity might also play a role, at least when Aß-signaling is predominant. Overall, and more speculatively, the clinical observations of abnormal tactile processing in ASD might be mostly related to the tactile features signaled by Aß-fibers. We believe that the interplay between intensity and pleasantness of touch should be taken into consideration in future studies.

**SUPPLEMENTAL MATERIAL: REFERENCES**

1 Smith, D. V. *et al.* Characterizing individual differences in functional connectivity using dual-regression and seed-based approaches. *Neuroimage* **95**, 1-12, doi:10.1016/j.neuroimage.2014.03.042 (2014).

2 Beckmann, C. F. & Smith, S. M. Probabilistic independent component analysis for functional magnetic resonance imaging. (2004).

3 Igelström, K. M., Webb, T. W. & Graziano, M. S. A. Neural Processes in the Human Temporoparietal Cortex Separated by Localized Independent Component Analysis. *J. Neurosci.* **35**, 9432, doi:10.1523/JNEUROSCI.0551-15.2015 (2015).

4 Igelström, K. M., Webb, T. W., Kelly, Y. T. & Graziano, M. S. A. Topographical Organization of Attentional, Social, and Memory Processes in the Human Temporoparietal Cortex. *eNeuro* **3**, ENEURO.0060-0016.2016, doi:10.1523/ENEURO.0060-16.2016 (2016).

5 Sohn, W. S., Yoo, K. & Jeong, Y. Independent component analysis of localized resting-state functional magnetic resonance imaging reveals specific motor subnetworks. *Brain Connect.* **2**, 218-224, doi:10.1089/brain.2012.0079 (2012).

6 Beissner, F., Schumann, A., Brunn, F., Eisenträger, D. & Bär, K. J. Advances in functional magnetic resonance imaging of the human brainstem. *Neuroimage* **86**, 91-98, doi:10.1016/j.neuroimage.2013.07.081 (2014).

7 Destrieux, C., Fischl, B., Dale, A. & Halgren, E. Automatic parcellation of human cortical gyri and sulci using standard anatomical nomenclature. *Neuroimage* **53**, 1-15, doi:<https://doi.org/10.1016/j.neuroimage.2010.06.010> (2010).

8 Fischl, B. *et al.* Automatically parcellating the human cerebral cortex. *Cereb. Cortex* **14**, 11-22, doi:10.1093/cercor/bhg087 (2004).

9 Filippini, N. *et al.* Distinct patterns of brain activity in young carriers of the APOE-ε4 allele. *Proc. Natl. Acad. Sci. U. S. A.* **106**, 7209, doi:10.1073/pnas.0811879106 (2009).

**SUPPLEMENTAL MATERIAL: FIGURE LEGENDS**

**Figure S1.** (A) Scatterplots showing Pearson’s correlations between the difference in ß-values for slow and fast touch and affective touch awareness scores in NS (*r* = 0.59, *p* < 0.004) and ASD (*r* = 0.006*, p* = 0.9). (B) Location of right STS mask used for local ICA (left) and the thresholded spatial map of the relevant IC (right; see Supplementary Methods) (C) Histograms from 100,000 bootstrap iterations computing correlations between pSTS response and affective touch awareness correlations. Frequency distribution for bootstrap iterations for ASD (gray, 95% CI [-0.25, 0.39]), for NS (white, 95%CI [0.22, 0.865) and for their respective difference (green, 95% CI [-0.94, -0.04]).

**SUPPLEMENTAL MATERIAL: TABLES**

**Table S1.** Activations associated with the whole-brain, gray matter within-group t-tests for slow and fast stimulation, expressed by peak scores in MNI space coordinates (x, y, z). Z-scores survived significance threshold (*p* < 0.002, cluster corrected alpha < 0.05). * indicate reverse contrast

| Analysis | Region | MNI  coordinates | | |  |
| --- | --- | --- | --- | --- | --- |
|  |  | x | y | z | voxels |
|  |  |  |  |  |  |
| **TD** |  |  |  |  |  |
| **Slow > Baseline** |  |  |  |  |  |
|  | Precentral Gyrus (Area 4) * | 40 | -23 | 67 | 122 |
|  | Supramarginal Gyrus (OP1) | -68 | -20 | 16 | 63 |
|  | Precentral Gyrus (Area 4) * | -23 | -29 | 61 | 60 |
|  | Supramarginal Gyrus | 49 | -32 | 28 | 44 |
|  | Postcentral Gyrus – lateral (Area 1) | 64 | -14 | 34 | 41 |
|  | Postcentral Gyrus – lateral (Area 2) | 28 | -41 | 55 | 32 |
|  | Cerebellum (Lobule VIII) * | -2 | -65 | -17 | 27 |
|  | Supramarginal Gyrus | -50 | -35 | 25 | 22 |
|  | Cerebellum (Lobule VIII) * | 1 | -68 | -32 | 19 |
|  | Precentral Gyrus (Area 4) * | -38 | -17 | 46 | 15 |
|  | Rolandic Operculum (OP1) | 52 | -20 | 19 | 13 |
|  | Posterior Insula | 40 | -14 | 13 | 11 |
|  | Supramarginal Gyrus (Area 1) | -62 | -23 | 43 | 11 |
|  | Posterior Insula | 37 | -17 | 19 | 10 |
|  | Middle Occipital Gyrus * | -29 | -86 | 31 | 8 |
|  | Insula | -41 | -5 | 16 | 7 |
|  |  |  |  |  |  |
| **Fast > Baseline** |  |  |  |  |  |
|  | Rolandic Operculum (OP1) / Posterior Insula | 64 | -20 | 16 | 140 |
|  | Precentral gyrus (Area 4) * | 43 | -20 | 64 | 93 |
|  | Postcentral gyrus (OP4) | -65 | -20 | 16 | 73 |
|  | Supramarginal gyrus | -50 | -38 | 25 | 46 |
|  | Precentral gyrus (Area 4) * | -23 | -29 | 64 | 38 |
|  | Postcentral gyrus (Area 2) | 28 | -41 | 55 | 35 |
|  | Precentral gyrus (Area 4) * | -38 | -17 | 43 | 19 |
|  | Posterior Insula | -41 | -17 | 16 | 18 |
|  | Supramarginal gyrus | 61 | -17 | 25 | 16 |
|  | Precentral gyrus (Area 4) * | 19 | -29 | 64 | 16 |
|  | Inferior Frontal gyrus | 43 | 43 | 7 | 14 |
|  | Insula | -41 | -5 | 16 | 12 |
|  | Insula | -44 | -5 | 1 | 11 |
|  | Precuneus * | 1 | -44 | 52 | 11 |
|  | Posterior Insula | 34 | -23 | 7 | 8 |
|  | Cerebellum (Lobule VIII) * | 4 | -65 | -35 | 8 |
|  | Cerebellum (Lobule VIII) * | 1 | -59 | -32 | 8 |
|  | Insula | 40 | 4 | -11 | 7 |
|  | Rolandic Operculum (OP4) | 55 | -5 | 7 | 7 |
|  |  |  |  |  |  |
| **ASD** |  |  |  |  |  |
| **Slow > Baseline** |  |  |  |  |  |
|  | Rolandic Operculum (OP4) | 61 | -20 | 16 | 232 |
|  | Supramarginal Gyrus (OP1) | -68 | -23 | 19 | 116 |
|  | Postcentral Gyrus (Area 1) | 37 | -41 | 67 | 115 |
|  | Precentral Gyrus (Area 6) * | 43 | -17 | 67 | 104 |
|  | Precentral Gyrus (Area 4) * | -29 | -26 | 73 | 66 |
|  | Supramarginal Gyrus | -62 | -23 | 40 | 46 |
|  | Cerebellum (Lobule VIII) | -14 | -71 | -47 | 42 |
|  | Postecentral Gyrus (3a) * | 19 | -29 | 61 | 36 |
|  | Cerebellum (Lovbule VI) * | 1 | -74 | -11 | 30 |
|  | Insula | -41 | -5 | 16 | 29 |
|  | Inferior Frontal Gyrus (Area 44) | -56 | 10 | 37 | 29 |
|  | Precentral gyrus (Area 4p) * | -38 | -17 | 43 | 23 |
|  | Insula (Area 13) | 43 | -11 | 10 | 22 |
|  | Frontal Operculum (OP3) | 40 | -2 | 16 | 21 |
|  | Superior Occipital gyrus (V2) * | 25 | -101 | 4 | 17 |
|  | Cingulate gyrus (Area 24) * | -2 | 1 | 43 | 16 |
|  | Rolandic Operculum (OP4) | 55 | -2 | 7 | 15 |
|  | Posterior Superior Frontal Gyrus (SMA) * | -2 | -20 | 58 | 13 |
|  | Cingulate gyrus (Area 24) * | -11 | -8 | 46 | 10 |
|  | Superior Temporal gyrus | 67 | -35 | 19 | 9 |
|  | Calcarine Gyrus (V1) * | 10 | -74 | 13 | 9 |
|  | Inferior Frontal Gyrus | 61 | 10 | 13 | 8 |
|  | Cerebellum (Lobule VI) * | -2 | -77 | -26 | 8 |
|  | Posterior Insula | 40 | -2 | -2 | 7 |
| **Fast > Baseline** |  |  |  |  |  |
|  | Retroinsular cortex | 46 | -32 | 25 | 155 |
|  | Medial frontal gyrus (Area 4) * | 1 | -29 | 76 | 153 |
|  | Precentral gyrus (Area 6) * | -29 | -23 | 76 | 138 |
|  | Parietal operculum (OP1) | -65 | -20 | 16 | 88 |
|  | Postcentral gyrus (Area 3b) | 31 | -38 | 70 | 80 |
|  | Precentral gyrus (Area 4) * | 46 | -17 | 61 | 69 |
|  | Precentral gyrus (Area 4) * | 22 | -26 | 61 | 38 |
|  | Postcentral gyrus - lateral (Area 1) | -65 | -20 | 34 | 21 |
|  | Lingual gyrus * | -2 | -65 | 7 | 21 |
|  | Postcentral gyrus - lateral (Area 1) | 61 | -14 | 37 | 19 |
|  | Inferior frontal gyrus (Areas 44) | -56 | 10 | 19 | 15 |
|  | Cerebellum (vermis VIII) * | -2 | -68 | -35 | 15 |
|  | Intraparietal sulcus * | 31 | -71 | 34 | 14 |
|  | Cerebellum (vermis VI) * | 1 | -65 | -17 | 12 |
|  | Precuneus * | -11 | -44 | 58 | 12 |
|  | Cerebellum (VIII) | -14 | -71 | -53 | 10 |
|  | Calcarine Gyrus (V1) * | 10 | -74 | 13 | 10 |
|  | Posterior insula (Area 13)* | -32 | -29 | 16 | 10 |
|  | Precentral gyrus (Area 6) * | -17 | -17 | 76 | 10 |
|  | Cerebellum (VIII) * | 28 | -44 | -53 | 9 |
|  | Insula | -35 | 1 | 10 | 7 |
|  | Prostcentral gyrus (Area 1) | 34 | -29 | 64 | 7 |
|  | Cuneus * | 13 | -74 | 34 | 7 |
|  | Precuneus * | 1 | -62 | 61 | 7 |
|  | Paracentral lobule * | 1 | -41 | 61 | 7 |
|  |  |  |  |  |  |
